# Generational status and duration of residence predict diabetes prevalence among Latinos: the California Men's Health Study

**DOI:** 10.1186/1471-2458-9-392

**Published:** 2009-10-19

**Authors:** Ameena T Ahmed, Virginia P Quinn, Bette Caan, Barbara Sternfeld, Reina Haque, Stephen K Van Den Eeden

**Affiliations:** 1 Division of Research, Kaiser Permanente, Oakland, USA; 2 Department of Research and Evaluation, Kaiser Permanente Southern California, Pasadena, USA

## Abstract

**Background:**

Diabetes disproportionately affects Latinos. However, examining Latinos as one group obscures important intra-group differences. This study examined how generational status, duration of US residence, and language preference are associated with diabetes prevalence and to what extent these explain the higher prevalence among Latinos.

**Methods:**

We determined nativity, duration of US residence, language preference, and diabetes prevalence among 11 817 Latino, 6109 black, and 52 184 white participants in the California Men's Health Study. We combined generational status and residence duration into a single migration status variable with levels: ≥ third generation; second generation; and immigrant living in the US for > 25, 16-25, 11-15, or ≤ 10 years. Language preference was defined as language in which the participant took the survey. Logistic regression models were specified to assess the associations of dependent variables with prevalent diabetes.

**Results:**

Diabetes prevalence was 22%, 23%, and 11% among Latinos, blacks, and whites, respectively. In age-adjusted models, we observed a gradient of risk of diabetes by migration status among Latinos. Further adjustment for socioeconomic status, obesity and health behaviors only partially attenuated this gradient. Language preference was a weak predictor of prevalent diabetes in some models and not significant in others. In multivariate models, we found that odds of diabetes were higher among US-born Latinos than US-born blacks.

**Conclusion:**

Generational status and residence duration were associated with diabetes prevalence among middle-aged Latino men in California. As the Latino population grows, the burden of diabetes-associated disease is likely to increase and demands public health attention.

## Background

Diabetes mellitus is a growing health problem among Latinos in the United States (US). Latinos have a higher prevalence and younger age of onset of diabetes than whites. Among California adults age 50-64, the prevalence of diabetes is 22.2% among Latinos, compared to 16.2% among African-Americans, 10.7% among Asians, and 8.1% among whites [1]. This disparity persists even after adjustment for obesity and socioeconomic status. As Latinos are the largest and fastest growing ethnic group in the US, constituting 14% of the population in 2006 and projected to constitute 24% by 2050, addressing diabetes among this ethnic group has huge public health importance [2]. Data that combine all Latinos into a single group, however, obscure significant differences that may exist within this population, particularly among strata defined by generational status, duration of residence in the US, or nativity.

Generational status and duration of US residence have been employed as proxy measures of acculturation. Acculturation has been defined as "those phenomena which result when groups of individuals having different cultures come into continuous first-hand contact, with subsequent changes in the original culture patterns of either or both groups."[3] Acculturation thus includes patterns of behavior and social interaction. While multi-item acculturation scales exist, proxy measures, including generational status, time since migration (for immigrants); and language use, a functional measure; have often been employed in the health literature [4]. As we acknowledge that acculturation is a multidimensional concept, we do not claim that our study findings necessarily be interpreted as evidence of the associations of acculturation with health outcomes. Rather, we consider the variables we employ to be indirect measures of exposure to US lifestyles, behaviors, and environment.

Explanations for ethnic disparities in diabetes prevalence include differential rates of physical inactivity and diabetogenic diet; obesity prevalence; genetic predisposition; socioeconomic status; and environmental factors. Few studies, however, have examined how generational status and duration of residence may explain disparities in diabetes prevalence *within *the Latino population or contribute to disparities *between *Latinos and other ethnicities. Latinos in the US are a very heterogeneous group, including recent immigrants whose patterns of health behavior and health status may markedly differ from those whose ancestors have lived in the US for multiple generations. Past cross-sectional studies of the associations between migration variables and diabetes prevalence had inconsistent findings. The purpose of this study was to determine how generational status, residence duration, and language preference are related to diabetes prevalence among middle-aged Latino men who participated in the California Men's Health Study (CMHS) cohort and to examine the extent to which these variables explain disparities in diabetes prevalence between Latinos, whites, and blacks.

## Methods

### Study Population

A description of the California Men's Health Study (CMHS) cohort, recruitment, and data collection methods has been reported previously [5]. Briefly, the cohort consists of 84 170 Northern and Southern California Kaiser Permanente (KP) members who completed mailed questionnaires in 2002 to 2003. The survey collected demographic characteristics (birthplace of respondent and his parents, and for immigrants, time since migration), health status, and life-style behaviors. Participants were men ages 45 to 69 years and who had been KP members for at least one year at the time of the first mailing. We have previously examined response bias and found that CMHS participants were similar to non-participant KP members of the same age. In particular, the prevalence of diabetes mellitus was nearly identical (13.5% for participants versus 13.6% for non-participants) [5]. We have also previously found the demographic characteristics of CMHS participants to be similar to those of male participants in the California Health Interview Survey, a population-based telephone survey of 55,000 California residents in 2001[6]. For the present study, we included only those respondents who reported their ethnicity as Latino, black, or white. We excluded 2075 CMHS participants who did not complete questions on ethnicity, birth place, or current weight and height. The cohort for the present analysis comprised 11,817 Latinos, 6109 blacks and 52,184 whites. Informed consent was obtained from all survey participants. This study was approved by the Institutional Review Boards of Kaiser Permanente Northern and Southern California.

### Measures of Ethnicity and Migration Status

CMHS allowed respondents to report one or more ethnicities. We defined as Latino all respondents who selected "Mexican, Central, South American, or other Hispanic," regardless of other ethnicities reported. We defined as white all respondents who selected "White-European" or "White-Middle Eastern" and did not also report Latino ethnicity. We defined as black all respondents who selected "Black or African-American" and did not also select Latino ethnicity. Among the 11,817 Latinos in our cohort, 7.6% also reported white ethnicity, 0.6% also reported black ethnicity (including 0.2% who reported black and white), and 2.2% reported other ethnicities. Country of origin was coded as U.S., Mexico, or other.

The survey assessed place of birth of the respondent and his parents, duration of residence in the US and language in which the respondent chose to take the survey. Generational status was defined as "immigrant" if a respondent reported birth outside the US, "second" if a respondent was born in the US and at least one parent was foreign-born, and "third or greater" if a respondent and both parents were US-born. We combined generational status and duration of residence into a single 6-category migration status variable, with levels: ≥ third generation; second generation; and immigrant living in the US for > 25, 16-25, 11-15, or ≤ 10 years. Language preference was defined as the language in which the study subject chose to complete the survey.

### Ascertainment of Diabetes

Diabetes was defined as survey self-report of diabetes mellitus or inclusion in the KP Diabetes Registries. The registry, for which methods have been described previously, has served as the basis for numerous epidemiology and health services studies [7-15]. In brief, registry eligibility is based on multiple sources of data including diabetes medication prescriptions, HbA1C levels, and outpatient, emergency room and hospitalization diagnoses. The registry was 99.5% sensitive for diagnosed diabetes, compared with self-report, as of January 2003.

### Assessment of Covariates

Covariates were self-reported on the CMHS survey. Covariates included age, income, educational attainment, body mass index (calculated from self-reported weight and height), physical activity (quartiles), alcohol consumption (quartiles), total daily calories (quartiles), and percent of calories from fat (quartiles). We chose these behaviors, as well as body mass index, because they are known to be associated with diabetes [16-18]. We assessed diet with a detailed semi-quantitative food-frequency questionnaire adapted from a questionnaire developed for the Women's Health Initiative and other studies, [19-21] modified to men's studies of prostate health [22]. We assessed physical activity with questions adapted from the CARDIA Physical Activity History (PAH) [23-25] that queried the men about the frequency and duration of their participation in specific types of moderate and vigorous recreational, household, and work-related activities. The CARDIA PAH has indirect validity against aerobic capacity and percent body fat [23,25] and a strong inverse relationship with most cardiovascular disease risk factors [26,27]. The CARDIA PAH provides summary scores in units of MET-minutes/week, derived by multiplying intensity of activity by frequency and duration, and summing over all activities. BMI was calculated from reported height and weight (weight in kilograms divided by the square of height in meters (kg/m^2^). We have previously compared BMI calculated from the CMHS self-reported data with BMI as recorded in medical records and found that over 80% of participants were classified into the same BMI category (i.e. normal weight, overweight, obesity class I, etc) by both sources, and 99.4% were classified into the same or adjacent categories [5].

### Analyses

We determined frequency of baseline characteristics among respondents of each ethnicity (Table 1), and among Latinos stratified by migration status and language preference (Table 2). Chi-squared or ANOVA was performed, as appropriate, to test for significant differences between groups. Logistic regression models were then specified to assess the association of migration status and language preference with prevalent diabetes among Latinos (Table 3). We first specified separate age-adjusted models of the effect of each variable on prevalent diabetes (Model 1). We then ran two additional sets of models in order to determine if significant differences were attributable to differences in socioeconomic status or health-related behaviors between groups. Model 2 controlled for age and socioeconomic status (income, educational attainment, employment status). Model 3 controlled for all variables of Model 2, as well as body mass index, physical activity, alcohol consumption, total daily calories, and percent of calories from fat.

**Table 1:** Baseline characteristics by ethnicity: California Men's Health Study.

	**Latino**	**Black**	**White**	**P value**
**Total (n)**	11 817	6109	52 184	

**Age (Mean, SD)**	57.2 (7.2)	58.8 (6.9)	58.7 (6.8)	< 0.0001

**Education**				< 0.0001
Less than high school	21.0	5.3	2.6	
High school or GED	18.2	15.8	10.7	
Vocational/technical school	7.8	4.9	4.1	
Some college	29.1	40.1	30.0	
College graduate	23.1	33.3	52.5	

**Language preference**				< 0.0001
Spanish	19.4	0.1	0.1	

**Acculturation index**				< 0.0001
1^st ^generation, < 10 years	1.5	0.4	0.3	
1^st ^generation, 11-15 years	2.6	0.3	0.4	
1^st ^generation, 16-25 years	10.1	1.7	1.3	
1^st ^generation, > 25 years	29.9	2.5	6.3	
2^nd ^generation	14.0	0.3	3.4	
3^rd ^generation or greater	41.9	94.9	88.4	

**Marital status**				< 0.0001
Married	82.9	73.5	81.2	
Never married	4.3	5.6	6.6	
Separated/divorced/widowed	12.3	20.3	11.9	

**Yearly household income ($)**				< 0.0001
< $20 000	9.2	5.5	3.1	
20 000-39 999	25.0	17.8	12.2	
40 000-59 999	21.9	21.2	17.6	
60 000-79 999	17.6	19.8	18.2	
80 000-99 999	9.9	13.5	14.2	
≥ 100 000	13.1	19.5	31.0	

**Body mass index (kg/m^2^)**				< 0.0001
< 25	16.4	19.1	25.7	
25-29	50.2	46.2	47.4	
30-34	23.8	24.5	19.5	
35-39	6.8	6.8	5.2	
≥ 40	2.7	3.5	2.3	

**Physical activity (METS-hours/week)**				< 0.0001
Q1 (< 320)	31.6	36.0	21.6	
Q2 (320-1040)	24.4	24.9	25.0	
Q3 (1041-2147)	22.0	18.8	26.6	
Q4 (≥ 2148)	21.4	19.8	26.6	

**Alcohol consumption (gm/day)**				< 0.0001
None	29.1	36.9	27.1	
Q1 (< 2.1)	25.9	22.3	15.4	
Q2 (2.1-8.2)	19.0	17.4	17.5	
Q3 (8.3-20.8)	14.3	13.2	19.7	
Q4 (≥ 20.9)	11.7	10.2	20.4	

**Total calories/day**				< 0.0001
Q1 (< 1447)	37.2	38.8	20.2	
Q2 (1447-1990)	23.0	22.8	25.8	
Q3 (1991-2618)	19.1	17.4	27.5	
Q4 (≥ 2619)	20.7	21.1	26.5	

**% calories from fat**				< 0.0001
Q1 (< 29.6)	24.1	22.5	25.6	
Q2 (29.6-34.9)	25.0	23.1	25.0	
Q3 (35.0-40.1)	26.7	26.6	24.9	
Q4 (≥ 40.2)	24.2	27.8	24.6	

**Hypertension**	40.1	59.5	40.4	< 0.0001

**Dyslipidemia**	64.8	67.4	67.0	< 0.0001

**Depression**	10.2	7.2	12.9	< 0.0001

**Current smoker**	15.4	18.2	9.8	< 0.0001

**Diabetes**	22.2	23.0	11.4	< 0.0001

With the exception of the first two rows (total number of subjects in each group, mean age with standard deviation), we present data as % of subjects of each ethnicity with the characteristic listed.

**Table 2:** Percentage distribution of participant characteristics by language preference and acculturation index among 11,817 Latinos: CMHS.

	**Language preference**			**Acculturation Index**						
			
				**1^st ^Gen-Years since migration**				**US-born**		
	**Span**	**Eng**	**P**	**< 10 y**	**11-15 y**	**16-25 y**	**> 25 y**	**2^nd ^Gen**	**≥ 3^rd ^Gen**	**P**

**Total (n (%))**	2296 (19.4)	9521 (80.6)		174 (1.5)	309 (2.6)	1189 (10.1)	3538 (29.9)	1648 (14.0)	4954 (41.9)	

**Age (Mean, SD)**	56.4	57.4	< .001	53.6	52.5	53.3	57.8	60.8	57.0	< .001

**Education**			< .001							< .001
Less than high school	55.6	12.6		21.8	30.1	41.3	34.9	12.4	8.3	
High school or GED	12.3	19.7		10.3	9.1	10.8	16.1	21.6	21.2	
Vocational/technical school	9.8	7.3		12.1	7.4	9.3	9.6	6.1	6.4	
Some college	8.1	34.2		12.1	14.2	15.7	20.6	35.6	37.9	
College graduate	12.3	25.7		42.5	37.5	21.3	17.7	23.6	25.8	

**Marital status**			< .001							< .001
Married	87.1	81.9		82.2	86.7	86.1	86.1	83.0	79.5	
Never married	2.2	4.8		5.8	2.6	3.6	2.9	4.3	5.5	
Separated/divorced/widowed	9.5	13.0		12.1	10.0	9.6	10.3	12.3	14.6	

**Yearly household income ($)**			< .001							< .001
< $20 000	23.1	5.85		25.9	17.2	17.1	12.2	6.2	5.0	
20 000-39 999	45.7	20.0		42.5	42.1	41.4	29.3	23.4	16.8	
40 000-59 999	19.3	22.6		20.1	24.6	20.9	22.9	21.5	21.7	
60 000-79 999	6.1	20.4		3.5	8.1	10.3	14.9	19.1	22.0	
80 000-99 999	1.9	11.9		4.6	3.2	4.3	7.6	11.5	13.1	
≥ 100 000	0.9	16.1		1.7	3.2	3.9	10.1	14.1	18.3	

**Body mass index (kg/m^2^)**			< .001							< .001
< 25	16.4	16.4		25.3	23.3	17.8	18.4	14.9	14.6	
25-29	53.6	49.4		52.3	52.1	53.2	53.8	49.9	46.8	
30-34	23.3	24.0		18.4	17.8	22.1	21.6	24.8	26.1	
35-39	5.1	7.3		3.5	4.5	5.3	4.6	7.7	8.8	
≥ 40	1.7	2.9		0.6	2.3	1.5	1.6	2.8	3.7	

**Physical activity (METS-hours/week)**			< .001							< .001
Q1 (< 320)	46.5	29.4		40.2	48.5	41.6	36.9	26.1	28.5	
Q2 (320-1040)	23.8	24.5		24.1	22.3	25.9	24.9	23.3	24.1	
Q3 (1041-2147)	16.1	22.5		17.2	15.2	16.7	20.2	24.1	22.7	
Q4 (≥ 2148)	11.1	23.4		16.7	13.3	13.4	17.0	26.2	24.5	

**Alcohol consumption (gm/day)**			< .001							< .001
None	28.3	29.3		27.6	25.6	29.1	26.4	30.5	30.9	
Q1 (< 2.1)	39.5	24.0		37.9	42.4	35.3	30.7	22.9	22.4	
Q2 (2.1-8.2)	18.2	18.8		22.4	19.1	19.3	19.7	16.4	18.4	
Q3 (8.3-20.8)	8.6	15.2		9.2	9.1	10.3	13.3	15.8	15.1	
Q4 (≥ 20.9)	5.4	12.7		2.9	3.9	6.0	9.9	14.3	13.3	

**Total calories/day**			< .001							< .001
Q1 (< 1447)	51.5	35.5		43.7	46.0	47.4	44.8	37.3	31.8	
Q2 (1447-1990)	19.1	23.4		25.3	21.7	19.5	21.5	22.5	24.1	
Q3 (1991-2618)	13.9	19.6		12.6	15.9	15.7	17.4	18.7	20.3	
Q4 (≥ 2619)	15.6	21.5		18.4	16.5	17.3	16.4	21.6	23.9	

**% calories from fat**			< .001							< .001
Q1 (< 29.6)	27.1	22.9		24.7	26.9	28.5	28.2	22.4	19.6	
Q2 (29.6-34.9)	27.1	24.5		34.5	27.8	27.1	28.7	23.8	21.8	
Q3 (35.0-40.1)	28.1	26.1		26.4	30.1	27.5	25.3	25.4	27.3	
Q4 (≥ 40.2)	17.6	26.5		14.4	15.2	16.9	17.8	28.4	31.4	

**Hypertension**	32.7	41.9	< .001	24.7	23.0	28.3	38.6	45.8	43.8	< .001

**Dyslipidemia**	59.7	66.0	< .001	53.5	58.9	57.4	65.7	68.0	65.7	< .001

**Depression**	11.4	10.0	.04	10.3	11.3	12.1	10.7	8.4	10.0	0.11

**Current smoker**	23.7	13.4	< .001	24.7	16.8	19.3	17.4	13.5	13.2	< .001

**Diabetes**	20.3	22.6	.02	12.1	13.3	16.7	21.3	25.5	23.8	< .001

Span: Spanish. Eng: English. Gen: Generation.

**Table 3:** Logistic regression models of prevalent diabetes mellitus, by acculturation among Latinos.

		**Model 1: Age-Adjusted**		**Model 2: Age, SES-Adjusted**		**Model 3: Full Model**	
	**N**	**OR (95% CI)**	**Ptrend**	**OR (95% CI)**	**Ptrend**	**OR (95% CI)**	**Ptrend**
**Acculturation Index**			0.0001		< 0.0001		0.09
≤ 10 y, imm	174	0.55 (0.35, 0.88)		0.49 (0.31, 0.79)		0.62 (0.38, 1.02)	
11-15 y imm	309	0.63 (0.45, 0.88)		0.58 (0.41, 0.83)		0.67 (0.46, 0.96)	
16-25 y, imm	1189	0.80 (0.67, 0.95)		0.70 (0.58, 0.84)		0.85 (0.70, 1.03)	
> 25 y, imm	3538	0.82 (0.74, 0.91)		0.77 (0.69, 0.87)		0.94 (0.83, 1.06)	
2^nd ^Gen ≥ 3^rd ^Gen	1648 4954	0.88 (0.77, 1.01) 1.0 (reference)		0.89 (0.78, 1.02) 1.0 (reference)		0.93 (0.81, 1.08) 1.0 (reference)	

**Language**			0.19		0.005		0.03
Spanish	2296	1.0 (reference)		1.0 (reference)		1.0 (reference)	
English	9521	1.08 (0.96, 1.21)		1.22 (1.0, 1.39)		1.17 (1.01, 1.35)	

Model 1 adjusts for age. Model 2 adjusts for age, income, and educational attainment. Model 3 (Full Multivariate Model) adjusts for age, income, educational attainment, body mass index, physical activity, alcohol consumption, total calories, and % of total calories from fat. Imm: immigrant.

In a final set of analyses (Table 4), we examined disparities in diabetes prevalence between US-born whites and blacks, and Latinos stratified by migration status. The purpose of these analyses was to examine how changes in the demographic distribution of Latinos, as the US-born population of Latinos grows, will affect diabetes prevalence compared to whites and blacks, who are largely US-born. We omitted foreign-born blacks and whites from analysis because small numbers in cells precluded meaningful analysis. Age was entered into multivariate models as a continuous variable. All other variables were entered as categorical variables. Migration status was entered into logistic regression models as a categorical variable. We employed the Proc Logistic function in SAS to evaluate evidence of a linear trend in risk of diabetes by acculturation index. Analyses were performed with SAS version 9.1 (SAS Institute, Cary, NC, USA).

**Table 4:** Adjusted models of prevalent diabetes mellitus, by acculturation among Latinos and among US-born blacks and whites.

		**Model 1: Age-Adjusted**		**Model 2: Age, SES-Adjusted**		**Model 3: Full Model**	
	**N**	**OR (95% CI)**	**Ptrend**	**OR (95% CI)**	**Ptrend**	**OR (95% CI)**	**Ptrend**

**US-born** **Whites**		1.0 (reference)	< 0.0001	1.0	< 0.0001	1.0	< 0.0001
**US-born** **Blacks** **Latinos**		2.37 (2.21, 2.53)		2.12 (1.97, 2.27)		1.82 (1.68, 1.96)	
≤ 10 y, imm	174	1.44 (0.91, 2.28)		1.15 (0.72, 1.83)		1.30 (0.81, 2.10)	
11-15 y imm	309	1.73 (1.24, 2.41)		1.42 (1.01, 1.99)		1.47 (1.03, 2.09)	
16-25 y imm	1189	2.17 (1.85, 2.54)		1.63 (1.37, 1.92)		1.81 (1.51, 2.15)	
> 25 y imm	3538	2.26 (2.07, 2.46)		1.80 (1.63, 1.98)		2.04 (1.84, 2.26)	
US-born	6602	2.66 (2.49, 2.83)		2.32 (2.17, 2.49)		2.17 (2.03, 2.33)	

Model 1 adjusts for age. Model 2 adjusts for age, income, and educational attainment. Model 3 (Full Multivariate Model) adjusts for age, income, educational attainment, body mass index, physical activity, alcohol consumption, total calories, and % of total calories from fat. Imm: immigrant.

## Results

### Cohort Characteristics

Characteristics of Latino, black and white CMHS respondents are presented in Table 1. The educational attainment and income of Latinos was much lower than that of whites; one fifth of Latinos did not graduate high school, and 34% had a household income less than $40,000. Obesity and overweight were common; only 16% of Latinos had a BMI less than 25. Among Latinos, 22% had diabetes mellitus, twice the prevalence among whites but nearly the same (23%) as among blacks.

Table 2 presents characteristics of Latino participants by generational status, duration of residence, and language preference. Nearly half of Latinos were immigrants, most of whom immigrated to the United States more than 25 years ago. English was the preferred language of 80%. Prevalence of diabetes increased with generational status and duration of residence (range, 12.1 to 23.8%), while English language preference was associated with only a small difference in diabetes prevalence (20.3% vs. 22.6%). There was a strong income gradient by generational status, duration of residence and language preference, with 5% of third generation men earning less than $20,000 year compared to 25% of those who immigrated within the past 10 years. These income frequencies by language were 6% and 23% for English and Spanish speakers, respectively. A similar gradient was observed in educational attainment, with US-born subjects more likely to have graduated high school than immigrants. Only 13% of English speakers were not high school graduates, compared to almost 56% of those who preferred Spanish. The percentage of college graduates was greatest among those who immigrated in the past 10 years (42.5%) and decreased among immigrants with longer time since migration.

In age-adjusted models, we observed a gradient of risk of diabetes with increasing levels of migration status (Table 3). Additional adjustment for socioeconomic status increased the magnitude of effect. Further adjustment for obesity and health behaviors accounted for some of the migration-related differences in diabetes prevalence, but the same gradient of risk across migration status remained, albeit with a borderline test for trend (p = 0.09). Language preference was not significantly associated with prevalent diabetes in age-adjusted models, (OR = 1.08, 95% confidence interval (CI) 0.96, 1.21, p = 0.19), though it was associated with a modest increased risk in models that additionally controlled for SES, obesity, and health behaviors. When migration status and language preference were entered into multivariate models together, language preference was no longer a significant predictor, and the magnitude and significance of effect of migration status on diabetes prevalence was no different than in models without language preference. Hence, we present models of migration status and language preference separately in Table 3.

We then ran a series of models to examine the extent to which migration status explains disparities between Latinos, blacks, and whites in diabetes prevalence (Table 4). We found that odds of diabetes were higher among US-born Latinos than US-born Blacks, as compared with US-born whites, in all models (p < 0.01 for Model 1, p = 0.03 for Model 2, p = 0.001 for Model 3). In the full multivariate model, US-born Latinos had an OR 2.17 (95% CI 2.03, 2.33) while US-born blacks had an OR 1.82 (95% CI 1.68, 1.96). Further, we found that in our full model, those Latinos with the shortest duration of residence, immigrants in the US < 10 years, had an OR 1.3 (95% CI 0.81, 2.10), which was not significantly different from US-born whites. This lack of statistical significance may be due to the small number of men (n = 174) in the < 10 year group.

## Discussion

We found that migration status (i.e. higher generational status and longer duration of US residence) was associated with prevalence of diabetes among middle-aged Latino men in California. These differences were partially attributable to increases in obesity and diabetogenic diet (i.e. more calories consumed and higher percent of calories from fat) with higher migration status, although the test for trend was of borderline significance. This suggests that other, unmeasured factors are also responsible for the observed association between migration status and diabetes prevalence. We observed a gradient of progressively increased risk with higher migration status. Language preference was a weak predictor of diabetes in some models, and not predictive in others.

There are several explanations for our finding that migration status is associated with diabetes prevalence. First, we found that obesity and diabetogenic diet increased among Latinos with higher migration status, and that adjustment for these variables partially explains the migration-associated risk for diabetes. Higher prevalence of obesity is a direct mechanism through which exposure to the US environment may lead to changes in behaviors that are associated with diabetes. However, our data suggest additional factors are at play since our results were robust, even after adjusting for BMI (and many other factors). As Latinos live in the US for a longer duration of time, culture shifts may occur, with consequent changes in dietary preferences and patterns. It is possible that other dietary factors, such as carbohydrate intake and glycemic index, may change with migration status and explain the differences we observed with migration status [28]. Other studies have similarly found that length of time living in the United States, but not language, are associated with obesity [29-33]. Second, acculturative stress, which is the stress associated with adjusting to a new social environment [34], or the stress associated with perceived discrimination against minorities and immigrants, may cause chronic physiological changes that lead to increased risk of diabetes [35-42]. The cumulative effects of years of acculturative stress might lead to increased risk of diabetes, which would explain an increase in diabetes prevalence with increased time lived in the US. Acculturative stress was associated with poorer health among 3012 Mexican-origin adults,[41] and with higher levels of anxiety and depression symptoms among 148 Latino college students [43]. It is possible that levels of stress, or cumulative exposure to stress, differs by migration status. If this is the case, acculturative stress might explain some component of differences in diabetes prevalence associated with migration status. As CMHS did not collect data on stress, we are unable to test this hypothesis. Third, it is possible that recent Latino immigrants who develop diabetes are more likely to return to their countries of origin, and thus may not have participated in the CMHS cohort. This bias, operating like the "salmon bias" which has been hypothesized to account for observed lower mortality among some groups of foreign-born than US-born Latinos, may similarly account for the lower diabetes prevalence observed among foreign-born than US-born Latinos[44]. Additionally, the healthy migrant effect, which is the concept that people who are healthy are able to migrate while those who are not healthy remain in their place of origin, might explain lower prevalence of diabetes among more recent migrants. As we only have data on men who were Kaiser Permanente members, and who were therefore living in the United States at the time of the study, we cannot evaluate either the healthy migrant effect or salmon bias as potential explanations for our findings.

The few cross-sectional studies to examine the association between acculturation, including proxy measures (nativity, generational status, duration of residence) and functional measures (language ability and preference) and diabetes prevalence among Latinos in the United States have had mixed results. NHANES 1999-2002 [45] and the San Antonio Heart Study [46,47] (SAHS, 1979-1982) found that low acculturation, as measured by language (NHANES) and functional integration, cultural values, and family attitude scales (SAHS), was associated with increased odds of diabetes. Proyecto VER, a study conducted among Hispanics in Arizona, found that lower generational status and Spanish language preference, as assessed with a Hispanic Health and Nutrition Examination Survey (HHANES)-based scale, was associated with higher diabetes prevalence [48]. A study of gestational diabetes found that foreign birth was associated with lower prevalence of gestational diabetes among Mexican-American women but higher prevalence among women from Puerto Rico and Central and South America [49]. It is likely that some of the divergence in study findings is due to cohort effect. Data from the San Antonio Heart Study were collected in the 1980s, when the rates of obesity and diabetes in the United States were much lower than at present. The culture to which immigrant Latinos adapt, including normative diet and physical activity behaviors, has changed in the past 20 years. It is also possible that the divergent findings of previous studies were due to the use of different measures.

Our study has several strengths. We present contemporary (2002-2003) data on a large sample of Latino men in a state with one of the largest Latino populations. Our study sample is drawn from an insured population and is representative of the general population, with the exception of the indigent. Further, our population has uniform access to care, so findings are less likely to be biased by underascertainment of diabetes among those of low socioeconomic status, as is the case in the general population.

This study has several limitations. Our findings cannot necessarily be generalized to women. Gender differences in patterns of obesity associated with acculturation [30,47] may translate into gender differences in associations between migration and diabetes. The CMHS was not designed primarily to examine migration, and we were limited to what measures were collected, which allow us only a linear measure of migration status. The concept of acculturation more fully captures the process by which immigrants' values, behaviors, and beliefs may encompass both those of their country of origin as well as those of the country to which they migrate. Acculturation is a multifaceted construct, including language use, orientation towards Latino or mainstream US culture, and behaviors or preferences in friends, music, diet, and other life domains. These behaviors and attitudes in turn impact lifestyle risk factors, including exercise and diet. Ideally, research into how migration affects health would employ multidimensional measures [50] and validated scales of acculturation and related concepts [51-54]. Despite this limitation, we found clear monotonic trends in the association between our measures of migration status and diabetes, even after adjustment for socioeconomic status and body mass index. If it is in fact the case that our proxy measure of migration status is not perfectly correlated with a validated measure of exposure to US environment and culture, this would have created a conservative bias in our analysis; that is, we would observe a weaker association (odds ratio closer to 1.0) than is true. Our measure of language preference was the language in which a respondent chose to take the survey. We did not have a measure of language ability or language preference in different situations. In addition, our study was cross-sectional and we had no information on duration of diabetes or assessment of language preference at the time of diabetes incidence. Finally, our study, as with all survey studies, relied on self-report of physical activity, diet, and alcohol consumption. If it were the case that accuracy of reporting varied by language preference or acculturation index, this would be a source of bias. However, there is no data to suggest differential reporting bias by level of acculturation. Further, our findings are in the same direction and of similar magnitude whether we employ the full multivariate model or the model adjusting only for age and socioeconomic status.

## Conclusion

Our findings have implications for public health and clinical medicine. We found a doubling in diabetes prevalence among Latinos who have been in the US over 25 years compared to those who immigrated within the past 10. The risk of diabetes was higher among US-born Latinos than US-born blacks in all models. On the other hand, the most recently immigrated Latinos had a risk of diabetes that was similar to that for US-born whites. As the US-born population of Latinos increases, this portends a worsening of the diabetes epidemic among Latinos to levels higher than the already alarming levels among blacks. Our finding that changes in obesity, diet, and physical activity only partially explain this elevated diabetes prevalence, however, points to the need for investigation into other factors that change with increased exposure to life in the US. For instance, the role of changes in particular dietary parameters, in acculturative or other stress, and in return migration to the country of origin as potential explanations for changes in diabetes prevalence deserve investigation. Further, studies should be conducted among women, as well as across the lifespan, to determine if explanatory factors vary across the life course. Research on diabetes and obesity among Latinos needs to account for migration status, as differences among populations may result in very different patterns of disease. We were unable to locate any prospective studies of the influence of migration status or measures of acculturation on diabetes incidence among Latinos. Prospective investigation of those factors responsible for the difference in risk between highest and lowest acculturated Latinos will be essential to stemming the problem of diabetes in the Latino community. It is possible that some of the health-related beliefs or practices among more recent Latino migrants might be leveraged in the design of interventions to prevent an increase in diabetes prevalence in Latino populations.

## Competing interests

The authors declare that they have no competing interests.

## Authors' contributions

ATA conceived of and conducted the analysis and drafted the manuscript. ATA, VPQ, BC, BS, RH, and SKV participated in critical reading and revision of the manuscript. All authors read and approved the final manuscript.

## Pre-publication history

The pre-publication history for this paper can be accessed here:


